# Scaffolding proteins in pediatric glioma

**DOI:** 10.18632/aging.203659

**Published:** 2021-10-26

**Authors:** Caroline Capdevielle, Martin Hagedorn

**Affiliations:** 1 Département de Biochimie et Médecine Moléculaire, CP 6128, Succursale Centre-Ville, Montréal, QC, H3C 3J7, Canada; 2 Université Bordeaux, Campus de Carreire, Victoire, Sciences de la Santé, Sciences de l'Homme, Bordeaux 33076, CEDEX, France; 3 Inserm U1035, Bâtiment TP Zone Sud, Bordeaux 33000, France

**Keywords:** scaffolding proteins, glioma, EBP50, IRSp53, Panobinostat

**Figure 1:**
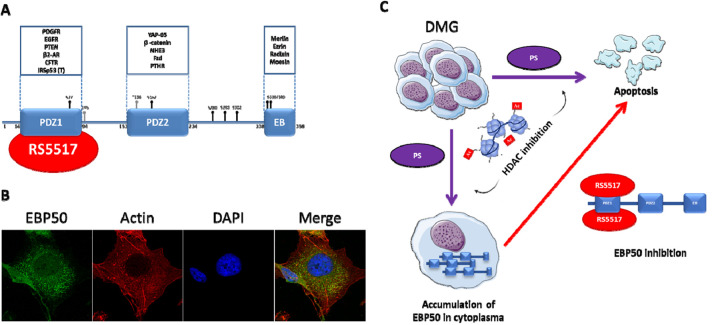
**EBP50 structure, localization and effect in DMG cell lines after Panobinostat treatment.** (**A**) EBP50 protein structure with PDZ and Ezrin Binding (EB) domains and possible interactions. Phosphorylation sites are also annotated. (**B**) EBP50 localization in DMG cell line treated with 1 µM of Panobinostat. EBP50 protein (in green) is found in all cellular compartments but in majority in the cytoplasm and the nucleus. (**C**) Panobinostat is a HDAC inhibitor which induces apoptosis in DMG cell lines. An accumulation of EBP50 is also observed in the cytoplasm of treated cells. Combinatory treatment with an inhibitor of EBP50, RS5517, leads to enhanced cell death.

Fundamental biological processes such as cell division and migration are important during lifetime, from very early embryonic development on up to aging individuals. Proteins regulating these events may play different roles regarding the biological age of a human. However, certain functional aspects of molecular regulators may be conserved throughout lifetime. This is due to physical interactions between proteins and concertation of key organizers such as scaffolding proteins which bring certain important signaling molecules in contact which each other to control these processes [1]. As this is true in normal functioning cells, in pathological conditions such as cancer, interactions between scaffolding proteins and target molecules are altered and physiological processes are deregulated [2] (see Figure 1A).

In recent years, it has become clear that one important underlying tumor-initiating mechanism in diffuse midline glioma (DMG, formerly called DIPG) is a histone mutation (H3K27M) [3], which perturbs the epigenetic landscape in glial cells / oligodendrocytes. The H3K27M mutation can occur in all living organisms, from plants to higher eukaryotes [4]. It is a mutational hotspot which can modify overall gene expression patterns. These findings have initiated studies using epigenetic modifying drugs to overcome this imbalance and to used it as therapeutic agent in cancer [5].

To study epigenetic treatments effects on DMG cells, one of the most aggressive cancers, we used a label-free quantitative proteomic approach. Our results show that chemical HDAC (Histone deacetylases) inhibition using Panobinostat (LBH-589, Farydak^®^), a drug in clinical trials, induces selective expression of only two proteins, in three primary DMG cells. Both are scaffolding proteins, EBP50 (Ezrin-Radixin-Moesin Binding Phosphoprotein-50) and IRSp53 (Insulin Receptor Substrate Protein Of 53 KDa) [6]. This very selective association suggests a regulation of these scaffolding proteins by HDAC.

EBP50 upregulation after HDAC inhibition (HDACi) is also found in normal cells, several microarray studies carried out after HDACi in mesenchymal stromal cells for example point in the same direction [7]. Interestingly, in the same study, the list of genes induced by HDACi also contained BAIAP2, encoding for the IRSp53 protein. It may therefore be that these two proteins constitute a signaling hub initiated by HDACi.

The biological role of one of these proteins, EBP50, is rather complex. In fact, various studies have shown that pro or anti-tumor effects of this protein depend on its cellular compartment localization. When located at the membrane, EBP50 interacts with other proteins compared to cytoplasmic or nuclear expression. EBP50 expression close to the cell membrane reduces signaling activity of the PDGFR-PTEN-AKT pathway, thereby acting as a tumor suppressor. When expressed in the cytoplasm or nucleus however, it can interact with proteins such as β-catenin, and enhances their activity, leading to pro-tumoral cellular effects. As a result, its functions are highly dependent on context and cellular localization and vary between different tissues and cancer types [1]. In DMG, EBP50 seems to have pro-tumor effects based on its cytoplasmic and nuclear localization (see Figure 1B) and the cytotoxic effect of its inhibition.

Indeed, interfering with EBP50 can have therapeutic benefits in pathologies such as DMG. In order to understand the impact of EBP50 expression in DMG, we investigated the effect of siRNA-mediated depletion of the protein. This has resulted in a decrease in cell proliferation associated with an increase of apoptotic cells. Dual treatment with PS and siRNA against EBP50 also suggests a synergistic effect in reducing cell survival (see Figure 1C).

A chemical inhibitor of PDZ1 domain activity of EBP50 termed RS5517 [8] also shows anti-tumor effects in DMG. Results obtained with the RS5517 molecule in DMG cells indicate that a cellular redistribution of EBP50 or the blocking of PDZ domains rather than an expression inhibition would allow better control of the effects of the EBP50 protein. Indeed, it is involved in signaling pathways important to the survival of healthy cells. It is therefore preferable to limit its interactions rather than entirely block its expression for its use in targeted therapy.

In conclusion, the various roles of EBP50 reflect complex mechanisms of action. Its functions do not depend only on interactions with other proteins, but also on its location and phosphorylation sites which cause either its relocation or a change in affinity for certain proteins.
